# Transformation efficiency and formation of transformation products during photochemical degradation of TCE and PCE at micromolar concentrations

**DOI:** 10.1186/2052-336X-12-16

**Published:** 2014-01-08

**Authors:** Sina Dobaradaran, Holger Lutze, Amir Hossein Mahvi, Torsten C Schmidt

**Affiliations:** 1 The Persian Gulf Marine Biotechnology Research Center, Bushehr University of Medical Sciences, Bushehr, Iran; 2 Department of Environmental Health Engineering, Faculty of Health, Bushehr University of Medical Sciences, Bushehr, Iran; 3 Instrumental Analytical Chemistry, University Duisburg-Essen, Essen 45141, Germany; 4 Department of Environmental Health Engineering, School of Public Health, Tehran University of Medical Sciences, Tehran, Iran; 5 Center for Solid Waste Research, Institute for Environmental Research, Tehran University of Medical Sciences, Tehran, Iran

**Keywords:** Byproduct, Degradation, Photochemical, Trichloroethene, Photolysis

## Abstract

**Background:**

Trichloroethene and tetrachloroethene are the most common pollutants in groundwater and two of the priority pollutants listed by the U.S. Environmental Protection Agency. In previous studies on TCE and PCE photolysis and photochemical degradation, concentration ranges exceeding environmental levels by far with millimolar concentrations of TCE and PCE have been used, and it is not clear if the obtained results can be used to explain the degradation of these contaminants at more realistic environmental concentration levels.

**Methods:**

Experiments with micromolar concentrations of TCE and PCE in aqueous solution using direct photolysis and UV/H_2_O_2_ have been conducted and product formation as well as transformation efficiency have been investigated. SPME/GC/MS, HPLC/UV and ion chromatography with conductivity detection have been used to determine intermediates of degradation.

**Results:**

The results showed that chloride was a major end product in both TCE and PCE photodegradation. Several intermediates such as formic acid, dichloroacetic acid, dichloroacetaldehyede, chloroform, formaldehyde and glyoxylic acid were formed during both, UV and UV/H_2_O_2_ treatment of TCE. However chloroacetaldehyde and chloroacetic acid were only detected during direct UV photolysis of TCE and oxalic acid was only formed during the UV/H_2_O_2_ process. For PCE photodegradation, formic acid, di- and trichloroacetic acids were detected in both UV and UV/H_2_O_2_ systems, but formaldehyde and glyoxylic acid were only detected during direct UV photolysis.

**Conclusions:**

For water treatment UV/H_2_O_2_ seems to be favorable over direct UV photolysis because of its higher degradation efficiency and lower risk for the formation of harmful intermediates.

## Background

Presence of volatile organic compounds (VOCs) in water is an important concern to all who use groundwater as a source for different purposes. These compounds such as chlorinated aliphatic hydrocarbons (CAHs) can enter the water sources and endanger human health in a direct or indirect manner. TCE and PCE are two of the most frequently detected groundwater contaminants and have been found from different sources in widespread areas [1-3].

The U.S. Environmental Protection Agency (EPA) has classified TCE and PCE as priority pollutants on the basis of their possible carcinogenicity, widespread contamination and potential formation of vinyl chloride (VC) during anaerobic bioconversion [4-7]. Most conventional treatment processes such as coagulation, sedimentation, precipitative softening, filtration and chlorination are not efficient in removal of TCE and PCE. Other conventional treatment processes such as adsorption on activated carbon and air stripping are effective in removing these compounds from contaminated water but the contaminants are transferred to another phase, and the residuals still need to be treated [8].

On the other hand, advanced oxidation processes (AOPs) can be considered as efficient complimentary techniques to the conventional treatment processes and may completely destroy toxic organics such as TCE and PCE [9-12]. AOPs such as UV/H_2_O_2_ produce highly reactive species such as OH° that react rapidly with electron-rich organics and destroy most organic chemical compounds. Under specific conditions, AOPs could lead to complete mineralization of parent contaminants to water, carbon dioxide and mineral acids. The direct photolysis of TCE and PCE has proven to be effective to destroy these compounds in both aqueous and gas phases [13-17].

It was reported that dichloroacetyl chloride was the major primary product in the gas phase photolysis of TCE that in comparison to TCE is more toxic and significantly less degradable. Dichloroacetic acid, chloroform, methylene chloride, phosgene and trichloroacetyl chloride were other reported intermediates [14]. Aqueous phase photolysis of TCE and especially photolysis in combination of chemical has not received as much attention as gas phase photolysis. Chu and Choy [18] measured the photodegradation rate of TCE in surfactant micelles at 254 nm and reported enhancement of TCE degradation rate due to surfactant addition. During TCE degradation no chlorinated intermediates formation was reported. In another study, the direct UV photolysis of a 58 ppm TCE was studied. In this study 80% mineralization of TCE solution occurred within 40 min [19]. Hirvonen et al. [20] measured the removal efficiency of TCE using a low pressure mercury lamp at 254 nm and reported it as only 20% of that found for UV photolysis in combination with hydrogen peroxide. In a recent study, Li et al. [21] studied the formation of byproducts and kinetic modeling of TCE during direct photolysis. They reported the chloride ion as major end product and some compounds such as formic acid, di- and monochloroacetic acids, glyoxilic acid, oxalic acid, formaldehyde, mono- and dichloroacetylene and dichloroacetaldehyde as byproducts. In another study, UV photolysis and UV/H_2_O_2_ degradation of TCE was examined by Hirvonen et al. [22]. They found chloroacetic acids as byproducts by either direct photolysis or UV/H_2_O_2_. Mertens and Sonntag measured the photolysis of PCE in aqueous solutions at 254 nm, they reported chloride ions, carbon dioxide as end products and trichloroacetic acid, dichloroacetic acid and hypochlorite as the major byproducts [23]. In another study Yamada and Tsuno have reported a higher pseudo-first order rate constant for PCE in comparison to TCE during UV photolysis [24].

During the degradation of TCE and PCE via UV or UV/H_2_O_2_ some harmful intermediates such as haloacetic acids (HAAs) and chloroform may be formed. These compounds are known as disinfection byproducts (DBPs) in chlorination; therefore, they are strictly monitored during water treatment. For example, the total level of HAAs should not exceed 60 μg/L as the regulatory standard set for drinking water quality by EPA.

Most of the mentioned studies deal with the process efficiency for TCE and PCE removal and degradation byproducts using millimolar concentrations. With regard to concentration ranges of TCE and PCE in previous studies exceeding environmental levels [3] the present study deals with the degradation efficiency of TCE and PCE at five different micromolar concentrations with direct UV photolysis and UV/H_2_O_2_, identify the possible harmful byproducts and their concentrations at different initial content levels and compare the UV and UV/H_2_O_2_ processes in treatment of contaminated water by TCE and PCE.

## Methods

### Reagents

Trichloroethene (≥ 99.5%), tetrachloroethene (≥ 99.5%), formic acid (98%), chloroacetic acid (99%), dichloroacetic acid (99.2%), formaldehyde, trichloroacetic acid (≥ 99%), oxalic acid (≥ 99%), glyoxylic acid (98%), chloroacetaldehyde, acetonitrile of HPLC grade (≥ 99.9%), perchloric acid (70%) all prepared from Sigma Aldrich, sulphuric acid (VWR, 95%) and chloroform (Aldrich, 99.8%) were used. All solutions were prepared using ultrapure water (resistivity 18.2 MΩ cm).

### Apparatus and analytical methods

All UV and UV/H_2_O_2_ experiments were conducted using a Merry-go-round reactor (manufactured by H. & Th. Schneider Glasapparatebau, Germany).

The photon flow entering the reactor from the 15 W low pressure mercury lamp was 60.1 μ einstein m^-2^ s^-1^ at 254 nm, as determined by atrazine actinometry.

In each UV experiment, 60 mL of TCE and PCE solution at different concentrations (3.8, 7.6, 76.1, 190.3 and 380.5 μM), were transferred into separate cylindrical quartz vessels, placed inside the reactor and illuminated with monochromatic UV lamps.

In UV/H_2_O_2_ experiments, a molar ratio of H_2_O_2_ to TCE and PCE equal to 200 was added to TCE and PCE solutions of different concentrations (as above).

The temperature inside the reactor was maintained at 25°C by a water bath and circulator (HAAKE F6).

Samples were taken from the quartz vessel at different predetermined reaction times (1, 3, 5, 10, 20, 40, 60 and 70 min).

The dissolved oxygen level and pH were immediately determined after each run by HACH HQ40d DO meter and Metrohm 827 pH meter, respectively.

GC/MS (Shimadzu-QP2010) with a splitless injector and a 30 m* 0.25 mm id* 0.25 μm fused silica capillary column (RxiTM–5 ms) using headspace solid-phase microextraction (SPME) was used to determine TCE, PCE, chloroform, chloroacetaldehyde and dichloroacetaldehyde. Helium 5.0 was used as carrier gas, one ramp temperature program was employed with an initial temperature of 40°C for 2 min, followed by a ramp of 5°C/min to 120°C and held for 2 min. Ion source, injection and interface temperatures were 200, 280 and 250°C, respectively.

The concentrations of chloride ion, oxalic acid, formic acid, di-, tri- and monochloroacetic acids were determined by ion chromatography (Metrohm 883 basic IC equipped with ion separation and conductivity detector, a Metrohm 863 compact autosampler, Metrosep A supp 4 column. An aqueous solution of carbonate/bicarbonate 1.7 mM NaHCO_3_ and 1.8 mM Na_2_CO_3_ has been used as eluent in isocratic mode).

Formaldehyde and glyoxylic acid were measured as dinitrophenylhydrazones using a Agilent 1100 HPLC/UV system (with C18 Reversed Phase column manufactured by Macherey- Nagel).

## Results and discussion

### Degradation kinetics of aqueous TCE and PCE solutions

Aqueous solutions with initial concentrations of TCE and PCE of 3.8, 7.6, 76.1, 190.3 and 380.5 μM were illuminated in direct photolysis and also in UV/H_2_O_2_ processes.

Linear regression of the logarithm of TCE concentrations versus reaction time allowed calculating the first-order reaction rate constants.

As presented in Table 1, an increasing degradation rate of TCE and PCE with decreasing initial concentration is observed. The first order degradation rate increases by a factor of 4 and 5 for TCE and 3.5 and 6.2 for PCE in UV and UV/H_2_O_2_ processes, respectively, when the concentration at t = 0 drops from 380.5 to 3.8 μM.

**Table 1:** **First order degradation rate constants of TCE and PCE at different concentrations, time of process and % removal in UV and UV/H**_**2**_**O**_**2**_**systems**

**Initial concentration (μM)**	**TCE & PCE**	**UV process**			**UV/H**_**2**_**O**_**2**_**process**		
		**k**_**1**_**(min**^**-1**^**)**	**Time of process (min)**	**Removal (%)**	**k**_**1**_**(min**^**-1**^**)**	**Time of process (min)**	**Removal (%)**
3.8	TCE	0.0847	40	100	0.1766	10	100
	PCE	0.0888	40	100	0.251	10	100
7.6	TCE	0.0519	60	100	0.1613	20	100
	PCE	0.0573	60	100	0.1915	20	100
76.1	TCE	0.0269	70	91	0.0816	40	100
	PCE	0.0392	70	97.54	0.0966	40	100
190.3	TCE	0.0235	70	88.36	0.0467	60	100
	PCE	0.0358	70	95.92	0.0549	60	100
380.5	TCE	0.0209	70	82.3	0.0348	70	95.8
	PCE	0.0254	70	88.66	0.0405	70	97.7

This is due to lower photon/contaminants ratio when the TCE and PCE concentrations are increased in the solution. When TCE and PCE start to be degraded and the byproducts simultaneously begin to be generated, the presence of byproducts in the solutions will retard the overall degradation of contaminants and also their byproducts.

A rapid initial degradation in the beginning of the processes, followed by a slower degradation rate, was found during the photodegradation of contaminants (in both UV and UV/H_2_O_2_ processes). Similar observations were also reported by other studies at diluted concentrations [25,26].

### Change of pH during the processes

**Figure 1:**
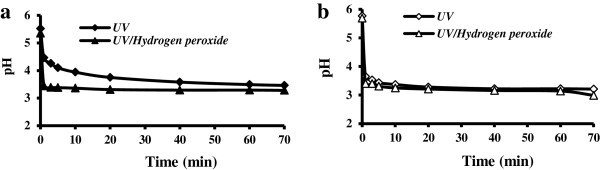
**pH change during TCE (a) and PCE (b) degradation in UV and UV/H**_**2**_**O**_**2**_**processes at initial concentration of 380.5 μM, molar ratio of H**_**2**_**O**_**2**_**to TCE and PCE equal to 200.**

The change of pH during irradiation in solutions at different initial TCE and PCE concentrations (3.8, 7.6, 76.1, 190.3 and 380.5 μM) was examined (in both UV and UV/H_2_O_2_ systems) with a similar initial pH of around 5.5. The variation of pH at highest initial concentrations of TCE and PCE is shown in Figure 1.

At low concentrations of contaminants (3.8 and 7.6 μM) variation of pH in solution was insignificant due to the small amount of protons produced in both systems (UV and UV/H_2_O_2_). At higher initial concentrations though, as shown in Figure 1(a-b), the initial pH of solutions drops sharply (especially in the UV/H_2_O_2_ system) after onset of irradiation (t?〈?3 min). The decrease in pH indicates that protons are generated as one of the major end products of the TCE and PCE photodegradation. The high amount of protons generated during UV irradiation retards degradation of PCE and TCE, so the overall reaction rates were lower at low levels of pH.

### Transformation products formation

Transformation products formed during irradiation of TCE and PCE at five initial different concentration levels (3.8, 7.6, 76.1, 190.3 and 380.5 μM) were examined. Intermediate formation will be discussed for the highest concentration (380.5 μM) with UV and UV/H_2_O_2_ processes.

**Figure 2:**
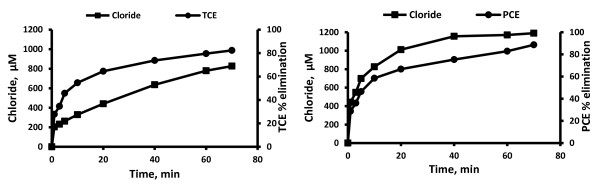
**Chloride formation and contaminants elimination by UV system at highest concentration (C**_**0**_**of TCE and PCE = 380.5 μM, t = 0 min).**

**Figure 3:**
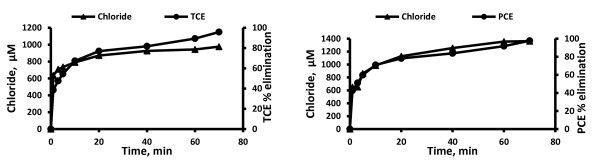
**Chloride formation and contaminants elimination by UV/H**_**2**_**O**_**2**_**system at highest concentration (C**_**0**_**of TCE and PCE = 380.5 μM, t = 0 min, molar ratio of H**_**2**_**O**_**2**_**to TCE and PCE equal to 200).**

**Figure 4:**
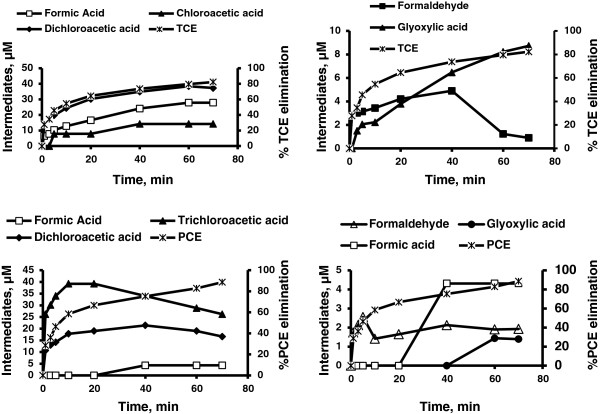
**Byproducts formation and contaminants elimination by UV system at highest concentration (C**_**0**_**of TCE and PCE = 380.5 μM, t = 0 min).**

**Figure 5:**
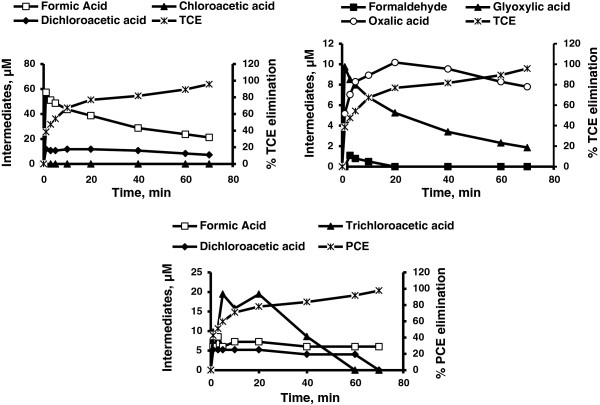
**Byproducts formation contaminants elimination by UV/H**_**2**_**O**_**2**_**system at highest concentration (C**_**0**_**of TCE and PCE = 380.5 μM, t = 0 min).**

For both TCE and PCE, chloride is a major end product. Several intermediates such as formic acid, dichloroacetic acid, dichloroacetaldyde, formaldehyde and glyoxylic acid were formed during both UV and UV/H_2_O_2_ treatment of TCE. However chloroacetaldyde, chloroacetic acid and chloroform were detected only during direct UV photolysis and oxalic acid was formed only during the UV/H_2_O_2_ system (Figures 2, 3, 4, 5 and Table 2). However, it is interesting to note that during UV/H_2_O_2_ process at lowest initial concentration of TCE (3.8 μM) low concentration of chloroform was detected (Table 3), but after 40 min, chloroform was removed completely. Formic acid, dichloroacetic acid and trichloroacetic acid were detected during both UV and UV/H_2_O_2_ treatment of PCE but formaldehyde and glyoxylic acid were only formed during UV photolysis (Figures 2, 3, 4, 5). Beside chloride as the major end product, formic acid and dichloroacetic acid were the main intermediates (with regard to their concentration) during both UV and UV/H_2_O_2_ treatment of TCE and for PCE di- and trichloroacetic acid were the main intermediates in both systems. However, the kinetics of formation and further transformation of those compounds during the processes were different (Figures 2, 3, 4, 5).

**Table 2:** **Transformation product formation (chloroacetaldehyde and dichloroacetaldehyde) and TCE elimination by UV and UV/H**_**2**_**O**_**2**_**systems at highest concentration (C**_**0**_**of TCE = 380.5 μM, t = 0 min, molar ratio of H**_**2**_**O**_**2**_**to TCE equal to 200)**

**Run time (min)**	**UV process**			**UV/H**_**2**_**O**_**2**_**process**		
	Chloroacetaldehyde (A)*	**Dichloroacetaldehyede (A)**	**% TCE elimination**	**Chloroacetaldehyde (A)**	**Dichloroacetaldehyede (A)**	**% TCE elimination**
1	0	70346704	27.76	0	838788	38.6
3	4573490	96891592	34.6	0	619849	47.58
5	3012691	135801181	45.72	0	323674	54.44
10	971776	164873121	54.7	0	218737	67.36
20	2305688	247528613	64.52	0	0	76.94
40	537962	187861453	73.7	0	0	81.76
60	429982	148046610	79.58	0	0	89.36
70	0	243483108	82.3	0	0	95.8

*Measured area via GC/MS.

**Table 3:** **Final concentrations of harmful transformation products during UV/H**_**2**_**O**_**2**_**processes of TCE & PCE and compare with EPA guidelines**

**Initial concentration of TCE & PCE**	**Contaminants**	**UV process**					**EPA guideline**					
		**Reaction time (min)**	**Concentration of contaminants and their transformation products after reaction time (μg/L)**				**TCE & PCE (μg/L)**		**HAAs (μg/L)**		**THMs (μg/L)**	
			**TCE/PCE**	**Dichloroacetic acid**	**Haloacetic acid**	**Chloroform(THMs)**	**MCL**^**a**^	**MCLG**^**b**^	**MCL**	**MCLG**	**MCL**	**MCLG**
3.8 μM (0.5 mg/L)	TCE	10	BLD	BLD	BLD^**^	0.6*	5	0	60	n/a	80	n/a
	PCE	10	BLD	BLD	BLD	BLD	Although there is no collective MCLG for this contaminant group, there are individual MCLGs for some of the individual contaminants: Trihalomethanes: bromodichloromethane (zero); bromoform (zero); dibromochloromethane (0.06 mg/L): chloroform (0.07 mg/L).					
7.6 μM (1 mg/L)	TCE	20	BLD	BLD	BLD	BLD						
	PCE	20	BLD	BLD	BLD	BLD						
76.1 μM (10 mg/L)	TCE	40	BLD	BLD	BLD	BLD						
	PCE	40	BLD	BLD	BLD	BLD						
190.3 μm (25 mg/L)	TCE	60	BLD	BLD	BLD	BLD	Haloacetic acids: dichloroacetic acid (zero); trichloroacetic acid (0.02 mg/L); chloroacetic acid (0.07 mg/L). Bromoacetic acid and dibromoacetic acid are regulated with this group but have no MCLGs.					
	PCE	60	BLD	BLD	BLD	BLD						
380.5 μM (50 mg/L)	TCE	70	2100	933.6	933.6	BLD						
		PCE	70	1150	BLD	BLD							BLD

*after 40 min was removed completely (BLD).

^**^BLD: Below detection limit.

^a^Maximum Concentration Levels.

^b^Maximum Concentration Levels Goal.

For TCE, the dynamics of the UV process suggests that chloride, formic acid, chloroacetic acid, dichloroacetic acid and glyoxylic acid were continuously generated.

Among these compounds, chloride, formic acid and dichloroacetic acid are formed at early irradiation stage in the UV system (Figures 2 and 4), but in the UV/H_2_O_2_ system just chloride was continuously generated (Figure 3). For PCE, the dynamics of the UV process showed that chloride, formaldehyde, di- and trichloroacetic acid were formed at early photolysis stage whereas formic acid and glyoxylic acid were formed only after 20 and 40 min, respectively (Figures 2 and 4). In contrast, in the UV/H_2_O_2_ system formic acid, di- and trichloroacetic acid were detected at early stages. After 60 and 70 min di and trichloroacetic acid were removed completely (Figure 5). The chlorine balance at the end of the experiment (70 min) indicates that for TCE approximately 12% and 10.7% less chloride than the initial level (~1141.5 μM, t = 0 min) in UV and UV/H_2_O_2_ systems respectively (with consider 82.3% and 95.8% TCE removal in UV and UV/H_2_O_2_ system respectively). For PCE, these values were 10.5 and 8.2% (~1522 μM, t = 0 min) in UV and UV/H_2_O_2_ systems respectively (with consider 88.6% and 97.7% PCE removal in UV and UV/H_2_O_2_ system respectively). This may be due to molecular chlorine, which should be among the inorganic species generated during the degradation and mineralization of chlorinated organics in UV and UV/H_2_O_2_ process. Hypochlorous acid (HOCl) was formed during the early stage of irradiation in both UV and UV/H_2_O_2_ systems. This compound will be formed from low levels of molecular chlorine dissolved in water [21].

HOCl is not a strong absorber of UV and the photolysis of this compound generates chlorine atoms and hydroxyl radicals, which may be further involved in degradation and oxidative reactions.

During light absorption, TCE and PCE degrade in aqueous solution by several major processes: (a) photo-stimulated hydrolysis, (b) homolytic cleavage of C-Cl bond, (c) loss of HCl, (d) loss of molecular chlorine.

When hydrogen peroxide is used in combination with UV light, photolysis of H_2_O_2_ leads to formation of more hydroxyl radicals in the system [27] so the destruction of contaminants and their byproducts will be faster and more efficient.

### Comparison of detected harmful transformation products levels during UV and UV/H_2_O_2_ with EPA standards

Chloroacetic acid, dichloroacetic acid and chloroform were detected during treatment of aqueous TCE solutions by direct photolysis and dichloroacetic acid was formed in UV/H_2_O_2_ process (also chloroform at the lowest initial concentration of TCE) (Table 4 and Table 3). For PCE, di- and trichloroacetic acids were detected in both UV and UV/H_2_O_2_ systems.

**Table 4:** Final concentrations of harmful transformation products during UV processes of TCE & PCE and compare with EPA guidelines

**Initial concentration of TCE & PCE**	**Contaminants**	**UV process**							**EPA guideline**					
		**Reaction time (min)**	**Concentration of contaminants and their transformation products after reaction time (μg/L)**						**TCE & PCE (μg/L)**		**HAAs (μg/L)**		**THMs (μg/L)**	
			**TCE/PCE**	**Chloroacetic acid**	**Dichloroacetic acid**	**Trichloroacetic acid**	**Haloacetic acid**	**Chloroform(THMs)**	**MCL**^**a**^	**MCLG**^**b**^	**MCL**	**MCLG**	**MCL**	**MCLG**
3.8 μM (0.5 mg/L)	TCE	40	BLD^*^	BLD	BLD	BLD	BLD	BLD	5	0	60	n/a	80	n/a
	PCE	40	BLD	BLD	BLD	BLD	BLD	BLD	Although there is no collective MCLG for this contaminant group, there are individual MCLGs for some of the individual contaminants: Trihalomethanes: bromodichloromethane (zero); bromoform (zero); dibromochloromethane (0.06 mg/L): chloroform (0.07 mg/L).					
7.6 μM (1 mg/L)	TCE	60	BLD	BLD	BLD	BLD	BLD	BLD						
	PCE	60	BLD	BLD	BLD	BLD	BLD	BLD						
76.1 μM (10 mg/L)	TCE	70	900	BLD	387.7	BLD	387.7	BLD						
	PCE	70	246	BLD	233.6	BLD	233.6	BLD						
190.3 μm (25 mg/L)	TCE	70	2910	1045	1851.8	BLD	2896.8	2.75	Haloacetic acids: dichloroacetic acid (zero); trichloroacetic acid (0.02 mg/L); chloroacetic acid (0.07 mg/L). Bromoacetic acid and dibromoacetic acid are regulated with this group but have no MCLGs.					
	PCE	70	1020	BLD	823.9	1433.7	2257.6	BLD						
380.5 μM (50 mg/L)	TCE	70	8850	1343	4807	BLD	6150	3.85						
	PCE	70	5670	BLD	2144.4	4283	6427.4	BLD						

^*^BLD: Below detection limit.

^a^Maximum Concentration Levels.

^b^Maximum Concentration Levels Goal.

As shown in Table 4 and Table 3, dichloroacetic acid reaches the highest concentration of all transformation products in both UV and UV/H_2_O_2_ processes during degradation of TCE.

Considering the concentrations of harmful transformation products in Table 4 and Table 3 it seems that the UV process is a promising treatment method only at low concentrations of TCE and PCE (0.5 and 1 mg/L).

In contrast, UV/H_2_O_2_ was a promising treatment method in removing contaminants also at higher concentrations.

Although at the highest initial concentration of TCE, high concentration of dichloroacetic acid was produced that exceeded the MCL levels of EPA standards. For PCE, although during UV/H_2_O_2_ process di- and trichloroacetic acids were formed but in the final reaction times, these compounds were not detected (Table 4 and Table 3).

## Conclusion

In summary, the initial degradation of TCE and PCE with UV and UV/H_2_O_2_ was more rapid in the beginning of the processes. It is confirmed in this study that initial concentration of TCE and PCE has an important role on the degradation rate constants and also generation of transformation products.

The use of direct UV irradiation when taking into account formation of harmful transformation products was successful only at low initial concentrations of TCE and PCE (3.8 and 7.6 μM).

TCE removal via UV/H_2_O_2_ was suitable at higher initial levels of TCE (3.8, 7.6, 76.1 and 190.3 μM) and for PCE this process (UV/H_2_O_2_) was suitable at all concentration levels in this study.

Finally, from these data it is concluded that HAAs formation from the photodegradation of TCE and PCE in raw water will be no problem in the UV disinfection of drinking water (considering their rather low environmental levels). But at higher concentrations (> 1 mg/L) for TCE and PCE degradation UV/H_2_O_2_ seems to be favorable over direct UV photolysis due to its higher degradation efficiency and lower risk for the formation of harmful intermediates.

## Competing interests

The authors declare that they have no competing interests.

## Authors’ contributions

SD has design and performed all experiments, analyzed data as well as wrote the manuscript. HL has guided the laboratory analysis, interpretation techniques and experiments structure. AHM has guided in the experiments design and manuscript preparation. TCS has designed the final version of experiments, final polishing and editing of manuscript as well as he was the supervisor of this study in all steps. All authors read and approved the final manuscript.
